# Two advanced-aged patients with diabetic striatopathy: a case report and literature review

**DOI:** 10.3389/fendo.2025.1598261

**Published:** 2026-01-19

**Authors:** Yikai Liu, Wentan Huang, Zewei Mo, Weiping Wei

**Affiliations:** 1 Department of Endocrinology, Hainan General Hospital, Hainan Affiliated Hospital of Hainan Medical University, Haikou, Hainan, China; 2 Bo’ao Town Community Health Center, Qionghai, Hainan, China

**Keywords:** blood glucose, diabetic striatopathy, hemichorea associated with ketotichyperglycemia, neostriatum, nervous system

## Abstract

**Background:**

Diabetic striatopathy (DS) is a rare neurological complication of diabetes mellitus, characterized by acute choreiform or ballistic movements and hyperintense basal ganglia lesions on T1-weighted MRI. While non-ketotic hyperglycemia is commonly implicated, DS can also occur in ketotic states.

**Case presentation:**

We report two elderly patients with long-standing, poorly controlled diabetes. Case 1, an 80-year-old male, presented with bilateral lower limb choreiform movements and delirium. Case 2, a 92-year-old female, developed right-sided hemichorea after discontinuing hypoglycemic therapy. Both patients had hyperglycemia with ketosis and characteristic basal ganglia T1 hyperintensity. Intensive glycemic control and symptomatic therapy led to complete resolution of involuntary movements in both cases.

**Conclusion:**

These cases highlight the heterogeneous clinical presentations of DS in advanced-aged patients and emphasize the importance of early recognition and prompt metabolic correction to achieve favorable neurological outcomes.

## Introduction

Diabetic striatopathy (DS) is a rare neurological syndrome associated with poorly controlled diabetes mellitus. Clinically, it manifests as acute or subacute chorea or hemiballismus, while neuroimaging typically shows hyperintense basal ganglia lesions on T1-weighted MRI. Although DS has traditionally been associated with non-ketotic hyperglycemia, emerging evidence indicates that ketotic hyperglycemia can also precipitate the condition. Advanced age, long-standing hyperglycemia, and cerebrovascular comorbidities may increase susceptibility of the basal ganglia to metabolic insults. Here, we present two elderly patients with DS triggered by ketotic hyperglycemia, with emphasis on clinical, laboratory, and neuroimaging characteristics, as well as outcomes following intensive management.

## Case 1

### Patient information

An 80-year-old man presented to the emergency department on December 4, 2024, with a 4-day history of acute agitation and altered consciousness accompanied by involuntary movements of both lower limbs. He was subsequently admitted to the endocrinology ward on December 5, 2024, for further evaluation and management. He had a documented 10-year history of type 2 diabetes mellitus and had previously been treated intermittently with insulin, although adherence and long-term glycemic monitoring were poor.

His medical history was notable for hypertension, heart disease, and prior intestinal surgery. There was no family history of similar neurological or metabolic disorders.

### Clinical findings

On arrival, the patient appeared emaciated and poorly nourished. He was uncooperative and provided irrelevant responses.

Vital signs were: temperature 36.2 °C, pulse 100 beats/min, respiratory rate 22 breaths/min, and blood pressure 142/86 mmHg.

Neurological examination showed grossly intact cranial nerve function, normal muscle tone, and full strength (grade V) in all limbs. Despite preserved strength, he exhibited involuntary choreiform movements predominantly affecting both lower extremities. Pulmonary auscultation revealed coarse breath sounds with moist rales. Cardiac examination was consistent with atrial fibrillation, with an irregular rhythm and a ventricular rate of approximately 108 beats/min. Skin examination demonstrated hyperpigmentation with scattered lesions over both lower limbs and pressure ulcers over the buttocks.

### Timeline

- Around December 1, 2024: Onset of acute agitation and delirium with progressive worsening, accompanied by involuntary movements of both lower limbs (per history).
- December 4, 2024: Emergency department evaluation revealed hyperglycemia and ketosis; initial symptomatic/supportive treatment was provided.
- December 5, 2024 (Hospital day 1): Admitted to the endocrinology ward; laboratory testing showed severe hyperglycemia (plasma glucose 30.63 mmol/L), markedly elevated HbA1c (18.19%), ketonuria, and increased D-3-hydroxybutyrate. Intravenous dextrose with low-dose insulin and potassium supplementation were initiated; haloperidol was started.
- December 6, 2024 (Hospital day 2): After resolution of ketonuria, continuous subcutaneous insulin infusion (CSII) was initiated. Neuroimaging demonstrated chronic cerebrovascular disease, and antiplatelet/cerebrovascular supportive therapy was added.
- December 10, 2024 (Hospital day 6): Marked improvement in choreiform movements, allowing gradual tapering of haloperidol.
- December 20, 2024 (Hospital day 16): Glycemic control stabilized with complete resolution of involuntary movements; patient discharged.

### Diagnostic assessment

At presentation, laboratory testing showed an elevated serum D-3-hydroxybutyrate level (4.55 mmol/L). Subsequent testing revealed severe hyperglycemia (30.63 mmol/L) and a markedly elevated HbA1c (18.19%). with ketonuria and increased serum D-3-hydroxybutyrate (2.36 mmol/L). Hematologic evaluation demonstrated leukocytosis (WBC 14.66 × 10^9^/L) with neutrophilia (90.1%). Biochemical testing showed elevated creatine kinase (1339.0 U/L) and B-type natriuretic peptide (950 ng/L). Thyroid function tests were largely within reference ranges, except for mildly reduced free triiodothyronine. C-peptide testing suggested impaired pancreatic β-cell function. Renal evaluation demonstrated proteinuria and microalbuminuria. Other virological, immunological, and biochemical tests were unremarkable according to available records.

**Figure 1:**
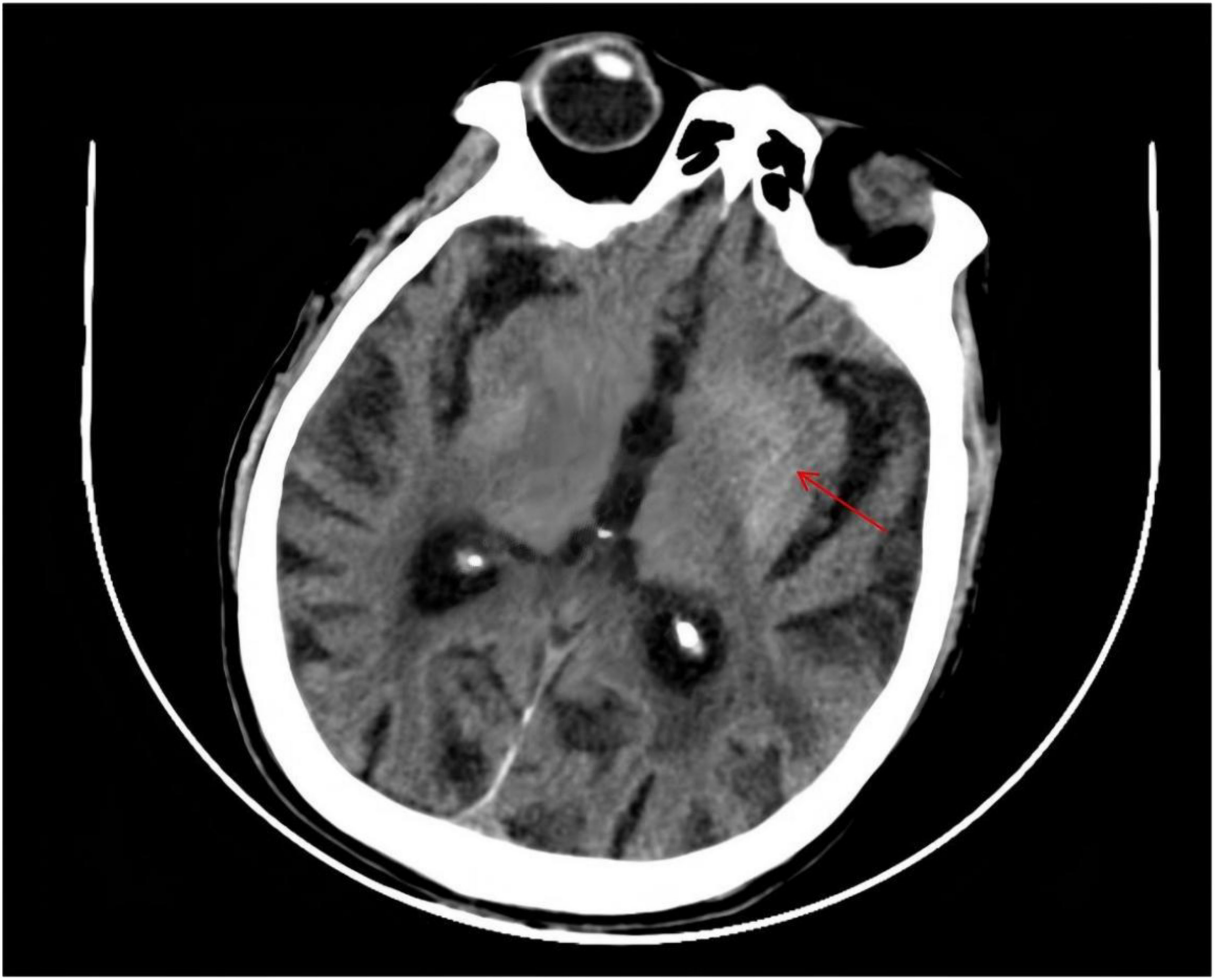
The CT scan reveals slightly low-density areas with a striped or linear configuration in the left parieto-occipital lobe, measuring approximately 3.0×3.6 cm.The CT value of the abnormal lesion center is approximately 36 HU. Abnormal signals were marked by red arrow.

**Figure 2:**
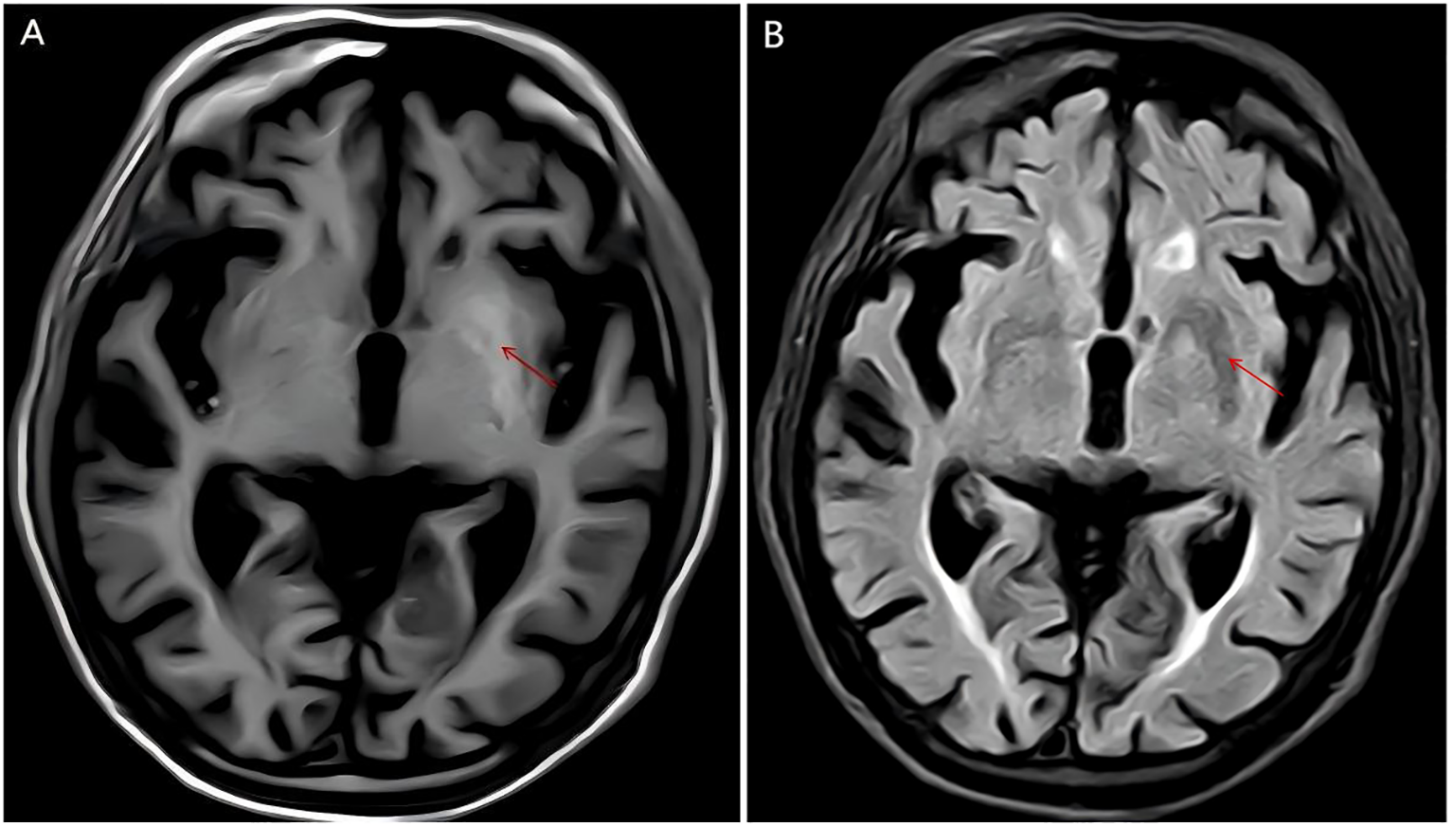
The MRI scan reveals patchy slightly shortened T1 signal foci in the bilateral basal ganglia regions, with ill-defined borders **(A)**. T2-weighted imaging (T2WI) and fluid-attenuated inversion recovery (FLAIR) sequences show low signals, more pronounced on the left side **(B)**. Abnormal signals were marked by red arrow.

Cranial CT demonstrated bilateral basal ganglia lacunar infarcts and diffuse cerebral atrophy (**Figure 1**). Brain MRI showed bilateral T1-weighted hyperintensity in the basal ganglia, more prominent on the left, compatible with DS (**Figure 2**). Additional findings included scattered lacunar infarctions and chronic cerebrovascular changes.

### Diagnostic synthesis

The differential diagnosis includes acute ischemic stroke and intracerebral hemorrhage; metabolic/toxic etiologies; drug-induced hyperkinesias, autoimmune/inflammatory disorders, infectious causes, and less commonly degenerative/genetic disorders such as Huntington disease. Given the patient’s established history of diabetes mellitus, the acute onset of choreiform involuntary movements, the presence of markedly uncontrolled hyperglycemia accompanied by ketosis, and the characteristic abnormalities involving the basal ganglia on brain MRI, a final diagnosis of DS associated with ketotic hyperglycemia was established.

### Therapeutic intervention

Management focused on correction of metabolic derangements and symptomatic control of involuntary movements. The patient received intravenous insulin with dextrose and potassium supplementation for ketosis correction and electrolyte management. After urinary ketones became negative, continuous subcutaneous insulin infusion (insulin pump therapy) was initiated for short-term intensive glycemic control, with basal rates and prandial boluses titrated according to bedside glucose monitoring.

Haloperidol (5 mg once daily) was administered for symptomatic control of choreiform movements and was tapered as symptoms improved. Bisoprolol fumarate was prescribed for ventricular rate control in atrial fibrillation, and atorvastatin calcium was initiated for lipid management.

Given concomitant cerebrovascular disease, neuroprotective and antiplatelet therapies were administered, including citidine diphosphate choline (0.2 g once daily), butylphthalide (0.2 g three times daily), ginkgo biloba extract (70 mg once daily), and clopidogrel (75 mg once daily). For cardiac insufficiency, treatment included isosorbide mononitrate sustained-release (40 mg once daily), trimetazidine (20 mg three times daily), and spironolactone (20 mg once daily), administered as clinically indicated. In addition, human albumin (50 g every other day) was infused to provide nutritional support.

### Follow-up and outcomes

Following metabolic correction and symptomatic therapy, the frequency and severity of involuntary movements decreased substantially. By December 10, 2024 (hospital day 6), choreiform movements had improved markedly, allowing gradual tapering of haloperidol. By December 20, 2024 (hospital day 16), glycemic control had stabilized and involuntary movements had completely resolved. The patient was discharged in stable condition with plans for ongoing diabetes management and follow-up for cardiovascular and renal comorbidities.

## Case 2

### Patient information

A 92-year-old woman was admitted to our hospital on December 22, 2024, for evaluation and management of 13 years of poorly controlled type 2 diabetes mellitus and a 6-day history of involuntary movements predominantly involving the right upper limb. She had a documented 13-year history of type 2 diabetes mellitus, diagnosed during a previous hospitalization, and had been maintained on metformin and gliclazide with irregular long-term glucose monitoring.

Several days before symptom onset, she discontinued her hypoglycemic medications, after which involuntary right arm movements emerged and progressively worsened. She was subsequently hospitalized at the Hainan Provincial Cadre Sanatorium, where she was diagnosed with diabetic striatopathy (DS). Despite six days of inpatient care, her involuntary movements persisted. She then presented to our emergency department on December 21, 2024, received supportive management, and was transferred to the Department of Endocrinology the following day for further treatment.

Past medical history: 13 years earlier, she had been diagnosed at our institution with (1) type 2 diabetes mellitus, (2) cerebral infarction, (3) facial neuritis, (4) hyperlipidemia, and (5) mild hepatic steatosis. Three days prior to the current admission, she was hospitalized at the Hainan Provincial Cadre Sanatorium with diagnoses including (1) type 2 diabetes mellitus, (2) multiple cerebral infarctions, (3) atherosclerotic coronary artery disease, (4) right lower-extremity fracture, (5) sinus tachycardia, and (6) grade 3 hypertension (very high risk). She reported no tobacco or alcohol use and no known drug allergies. Family history was notable for diabetes mellitus in her son.

### Clinical findings

On admission, vital signs were: temperature 36.2 °C, heart rate 113 beats/min, respiratory rate 20 breaths/min, and blood pressure 125/87 mmHg. The patient appeared moderately nourished and overweight and assumed a forced posture. She was conscious but largely unresponsive to questioning and was poorly cooperative during the examination.

No lower-extremity edema was present. Involuntary choreiform movements were observed in the right upper and right lower limbs, consistent with right-sided hemichorea. Because of limited cooperation, muscle strength and tone in the right extremities could not be reliably assessed. The left upper and left lower extremities demonstrated grade IV muscle strength with normal muscle tone. The left dorsalis pedis pulse was slightly diminished.

### Timeline

- Around December 16–21, 2024: Progressive involuntary movements of the right arm; later involving the right lower limb.
- December 21, 2024: Emergency department evaluation identified hyperglycemia with ketosis/ketonuria; supportive management initiated.
- December 22, 2024 (Hospital day 1): Transferred to and admitted in the endocrinology ward for further evaluation and treatment of suspected diabetic striatopathy.
- December 23, 2024 (Hospital day 2): Persistent right-sided choreiform movements prompted initiation of haloperidol; intensive glycemic control with CSII commenced.
- December 24–26, 2024 (Hospital day 3-5): Insulin pump regimen adjusted; dapagliflozin added, with subsequent stabilization of blood glucose levels.
- December 28, 2024 (Hospital day 7): Complete resolution of choreiform movements; haloperidol discontinued.
- December 30, 2024 (Hospital day 9): Discharged in good condition with stable glycemic control and no recurrence of symptoms.

### Diagnostic assessment

Emergency department laboratory testing (December 21, 2024) revealed blood glucose 17.14 mmol/L, serum D-3-hydroxybutyrate 1.82 mmol/L, positive urine ketones, and CRP 17.15 mg/L. She received initial supportive care with intravenous fluids and glucose-lowering therapy prior to transfer.

Post-admission testing showed improved but still abnormal metabolic indices, including blood glucose 9.53 mmol/L and D-3-hydroxybutyrate 0.59 mmol/L, with markedly elevated HbA1c 14.14% and glycated serum protein 2.46 mmol/L, indicating long-standing poor glycemic control. C-peptide testing demonstrated impaired endogenous insulin secretion (fasting C-peptide 0.330 nmol/L, postprandial C-peptide 0.647 nmol/L). Additional abnormalities included potassium 3.44 mmol/L, AST 44.3 U/L, LDH 306.8 U/L, α-hydroxybutyrate dehydrogenase 245.1 U/L, creatine kinase 612.2 U/L, CK-MB 28.9 U/L, BNP 583 ng/L, and high-sensitivity troponin T 0.016 μg/L. Nutritional markers suggested relative protein malnutrition (total protein 64.9 g/L, albumin 34.5 g/L, prealbumin 127.00 mg/L). LDL-C was 3.48 mmol/L, and 25-hydroxyvitamin D was decreased at 16.3 ng/mL. Erythrocyte sedimentation rate was markedly elevated (86 mm/h). White blood cell count was 9.96 × 10^9^/L. Other hepatic/renal indices and electrolytes were not clinically remarkable.

To exclude alternative etiologies, immunological and infectious screening—including diabetes-related autoantibodies, HIV and syphilis serologies, ceruloplasmin, anti-streptolysin O, rheumatoid factor, antinuclear antibodies (ANA), and anti–double-stranded DNA antibodies (IIF)—was negative.

**Figure 3:**
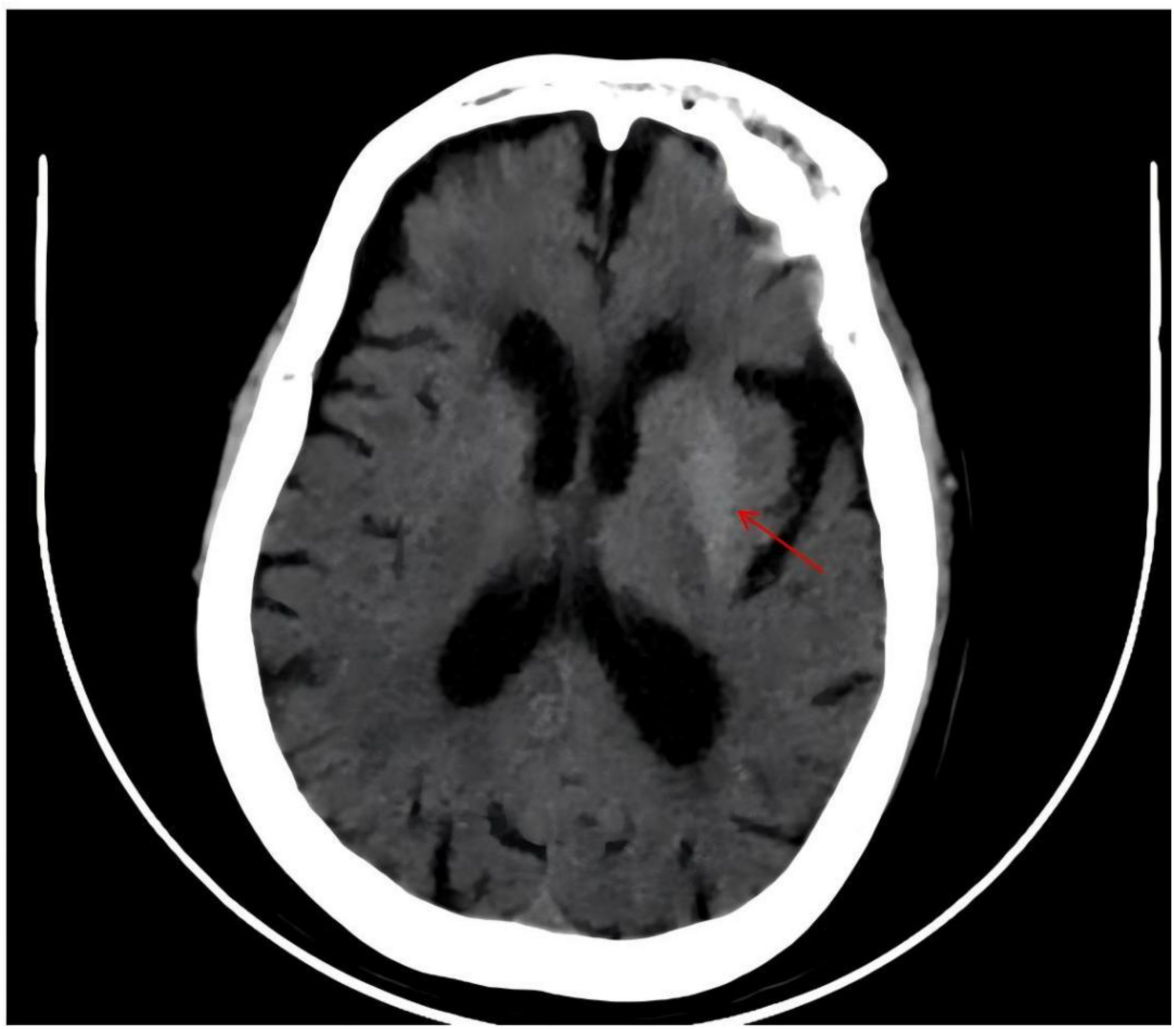
The CT scan reveals increased density in the left basal ganglia, with a lesion measuring approximately 3.5x1.1 cm, and a central CT value of approximately 33 HU. Abnormal signals are marked by red arrows.

**Figure 4:**
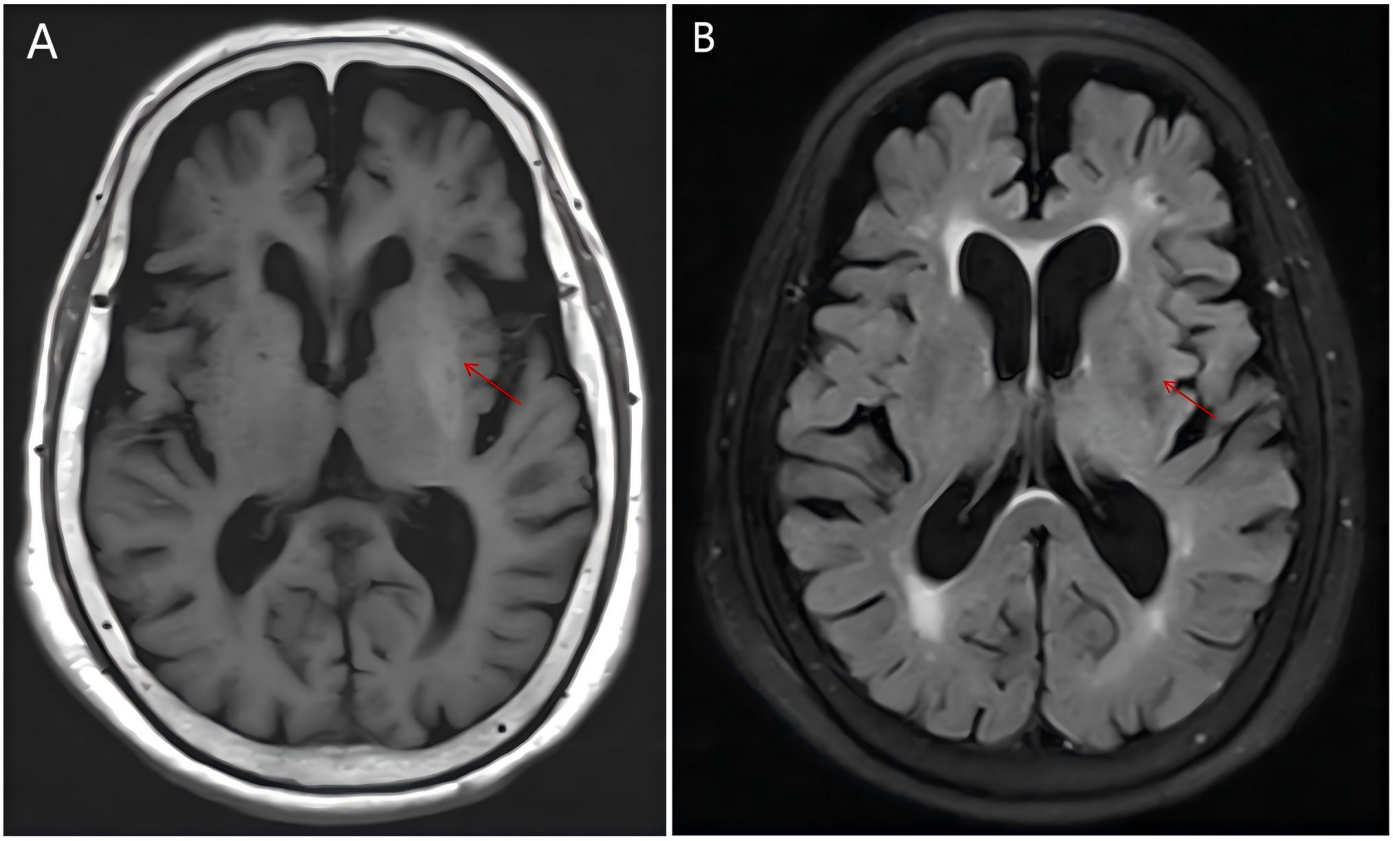
The MRI scan reveals patchy short T1 **(A)** and slightly short T2 signal shadows in the left basal ganglia **(B)**, with slightly high signal on the water suppression sequence, and no diffusion restriction. Abnormal signals are marked with red arrows.

Neuroimaging and vascular evaluation: Cranial CT: Hyperdensity in the left basal ganglia, with multiple lacunar infarcts in the bilateral frontoparietal lobes and corona radiata adjacent to both basal ganglia (**Figure 3**). Cranial MRI: Abnormal signal intensity in the left basal ganglia, interpreted as multiple vascular-origin cavitary lesions (lacunes). No acute cerebral infarction was identified. Bilateral periventricular white-matter hyperintensities consistent with Fazekas grade 1 and mild cerebral atrophy were present. MRA: Absence of the right anterior cerebral artery A1 segment and diffuse intracranial atherosclerosis, most prominent in the right middle cerebral artery (**Figure 4**).

### Diagnostic synthesis

The patient’s acute-right-sided choreiform movements in the setting of poorly controlled diabetes with hyperglycemia and ketosis, together with left basal ganglia hyperdensity on CT, supported the clinical diagnosis of diabetic striatopathy. Coexisting chronic cerebrovascular disease (lacunar infarcts and intracranial atherosclerosis) was considered a key confounder; however, the absence of acute infarction on MRI and the metabolic context favored DS as the unifying diagnosis.

### Therapeutic intervention

On December 21, 2024, in the emergency department, the patient received supportive care with intravenous fluids and glucose-lowering therapy and was transferred to endocrinology on December 22, 2024. After admission, inpatient management targeted metabolic stabilization and symptomatic control. Treatment included intravenous dextrose infusion with low-dose insulin and potassium chloride supplementation for electrolyte repletion, followed by continuous subcutaneous insulin infusion (insulin pump therapy) with frequent monitoring and protocol-guided titration.

Because right-sided involuntary movements persisted after transfer, haloperidol (5 mg once daily) was initiated on December 23, 2024, for symptomatic control. Metformin was reintroduced to optimize glycemic management. On December 24, insulin pump settings were adjusted (basal infusion increased to 22 U/day and prandial doses set at 8 U/meal). On December 26, dapagliflozin (10 mg once daily) was added, and prandial insulin was reduced to 7 U/meal.

For comorbid hypertension, dyslipidemia, coronary atherosclerosis, and prior cerebral infarction, she received: nifedipine sustained-release (30 mg once daily), atorvastatin (10 mg nightly), citidine diphosphate choline (0.2 g three times daily), clopidogrel (75 mg once daily), and bisoprolol fumarate (5 mg once daily).

### Follow-up and outcomes

With optimization of the glucose-lowering regimen, blood glucose remained stable and right-sided choreiform movements progressively improved. By December 28, 2024, involuntary movements of the right extremities had completely resolved, and haloperidol was discontinued. By December 30, 2024, glycemic control remained stable with no recurrence of involuntary movements, and the patient was discharged in good condition.

## Discussion

### Overview

Diabetic striatopathy (DS) is an uncommon neurologic complication of poorly controlled diabetes, seen predominantly in middle-aged and older patients, and characterized by acute or subacute choreiform movements, most commonly hemichorea (1). DS exhibits a diverse clinical presentation, primarily characterized by choreiform movements and systemic symptoms. The most salient feature of DS is hemichorea, which typically manifests as rapid, involuntary, and irregular movements confined to the ipsilateral upper and lower extremities, rarely involving both sides of the body. These movements are often described as “dance-like” due to their asynchronous and unpredictable nature, predominantly affecting distal limb segments. Systemic symptoms may include generalized weakness, cognitive impairment, anorexia, headache, and dizziness, reflecting the multifaceted impact of hyperglycemia on both neural and systemic homeostasis. The proposed pathophysiology involves metabolic derangements—particularly γ-aminobutyric acid (GABA) depletion and neuroinflammation—that disrupt basal ganglia circuits and produce hyperkinetic movements (2). Lateralized chorea may also involve facial and neck musculature, with accompanying neurological manifestations such as tongue abduction, unilateral brow furrowing, and bilateral buccal grimacing. These symptoms exhibit diurnal variability, often becoming more pronounced during periods of patient agitation or emotional stress, and may diminish or resolve spontaneously following sleep induction (3). DS can also coexist with cerebrovascular events and may be misdiagnosed as acute stroke; therefore, if abnormal movements abruptly subside while new focal neurological deficits emerge, clinicians should maintain a high index of suspicion for acute ischemic stroke and investigate urgently (4).

### Case summary

Our two patients shared the core elements of DS—markedly uncontrolled diabetes (HbA1c 18.19% in Case 1 and 14.14% in Case 2), acute/subacute choreiform movements, and supportive basal ganglia abnormalities on imaging—yet differed in clinically instructive ways.

First, both cases occurred in the setting of hyperglycemia with ketosis. This is clinically important because some clinicians implicitly equate DS with non-ketotic states and may discount DS when ketones are present. In our patients, the neurological course improved in parallel with metabolic stabilization, supporting the concept that hyperglycemia-driven striatal dysfunction can occur across ketotic and non-ketotic phenotypes (2).

Second, the motor phenotype differed substantially. Case 2 manifested as right-sided hemichorea, aligning with the classic DS phenotype. In contrast, Case 1 presented with bilateral lower-limb choreiform movements accompanied by delirium/altered consciousness, a pattern that may be underrecognized because it blurs the boundaries between movement disorder, metabolic encephalopathy, and vascular comorbidity. This heterogeneity underscores that DS should be considered even when the presentation deviates from classic unilateral HC–HB, particularly in advanced-aged individuals with reduced cerebral metabolic reserve (3).

Third, therapeutic interventions in both cases included intravenous rehydration, glycemic control protocols (6–9 mmol/L range maintenance), ketosis management, and dopamine receptor antagonists (haloperidol 2.5–5 mg/day), resulting in progressive resolution of choreiform movements. Continuous subcutaneous insulin infusion combined with oral hypoglycemic agents constituted the glycemic control strategy (**Table 1**). Both patients improved over several days, and involuntary movements resolved completely before discharge.

**Table 1:** Blood glucose monitoring and insulin therapy of the two patients after admission.

A											
Hospitalization	Blood sugar(mmol/L)							Insulin dose(IU)			
	Morning		Midday		Evening		bedtime	Morning	Midday	Evening	basal rate
	7:00	9:00	11:00	13:00	17:00	19:00	22:00				
D1	/	/	/	/	11.7	14.9	9.0	6	6	6	6
D5	6.8	/	5.9	7.4	/	17.3	19.1	4	4	4	5
D9	10.4	/	6.6	4.7	5.1	11.7	9.3	4	6	4	10
D13	7.2	4.6	8.7	9.8	12.0	10.7	9.8	6	6	6	18
D16	7.7	7.3	6.9	/	/	/	/	6	6	6	18

B											
Hospitalization	Blood sugar(mmol/L)							Insulin dose(IU)			
	Morning		Midday		Evening		bedtime	Morning	Midday	Evening	basal rate
	7:00	9:00	11:00	13:00	17:00	19:00	22:00				
D1	/	/	/	/	13.2	8.9	11.4	4	4	4	10
D3	14.7	20.2	14.8	14.1	12.2	16.1	13.1	8	8	8	22
D5	8.3	10.3	6.6	8.9	6.6	7.6	5.1	7	7	7	20
D7	7.7	10.8	9.0	7.7	6.3	6.4	4.6	6	6	6	18
D9	9.5	12.5	/	/	/	/	/	6	6	6	18

(A) represents case 1, 80 year old male patients, (B) represents case 2, 94 year old female patients. The symbol “/” indicates that the data is not applicable. Blood glucose is expressed in millimoles per liter (mmol/L), and insulin dosage is measured in international units (IU).

### Neuroimaging

DS is a rare hyperglycemic neurologic complication with characteristic neuroimaging findings. Radiological manifestations primarily involve bilateral basal ganglia structures, with predominant involvement of the putamen, head of the caudate nucleus, and globus pallidus. Cranial CT typically reveals unilateral or bilateral striatal hyperdensity within the basal ganglia region, which may be initially misinterpreted as cerebral hemorrhage requiring surgical intervention. However, these hyperdense lesions exhibit distinct differential features compared to conventional intracerebral hematomas, including absence of mass effect, limited lesion extent, heterogeneous signal intensity, and typically sparing of adjacent thalamic and internal capsule structures. These imaging features may correspond to localized microhemorrhages, dystrophic calcification, or transient mineral deposition associated with acute metabolic disturbances in hyperglycemic states (5).

In our series, CT attenuation values were modest (33–36 HU) compared with the ~40–50 HU often cited in DS (6, 7). This finding deserves explicit interpretation. Lower attenuation may reflect differences in scanner protocols, ROI selection/partial-volume effects, timing of imaging relative to symptom onset, or heterogeneity in underlying tissue composition. Importantly, a “not very dense” basal ganglia lesion should not dissuade clinicians from DS if the morphology and metabolic context are compatible.

On diffusion-weighted imaging (DWI), the lesions may exhibit mild hyperintensity or isointensity relative to surrounding parenchyma, while contrast-enhanced studies typically demonstrate no significant abnormal enhancement. Notably, the neuroimaging abnormalities observed in certain cases demonstrate potential reversibility, with gradual resolution of radiological findings correlating with effective glycemic regulation and amelioration of hyperkinetic symptoms. This neuroradiological normalization generally occurs within a temporal window of approximately 3 to 6 months following clinical symptom resolution, frequently exhibiting delayed imaging improvement compared to clinical recovery timelines (1).

While striatal abnormalities are identified on neuroimaging in most DS, important exceptions exist. Approximately 7% of patients whose predominant manifestation is chorea show no detectable lesions on computed tomography (CT) or magnetic resonance imaging (MRI), whereas about 2% exhibit radiologically evident striatal lesions without corresponding motor symptoms (8). Clinicoradiological lateralization may also be discordant: some patients demonstrate unilateral imaging abnormalities but bilateral chorea (9); others show bilateral striatal involvement with chorea confined to one side; and, in rare instances, symptoms occur ipsilateral to the radiological lesion (10). On the basis of these patterns, DS can be provisionally categorized into three phenotypes: (i) symptomatic DS (clinical chorea with supportive imaging), (ii) clinically isolated DS (clinical chorea without demonstrable striatal lesions), and (iii) radiologically isolated DS (striatal lesions in the absence of chorea) (11). These clinicoradiological discrepancies underscore the heterogeneity of DS and highlight the need for prospective studies to clarify their pathophysiological basis, temporal evolution, and implications for diagnosis and prognosis.

Conclusively, the cranial imaging characteristics of hyperglycemic lateralized chorea are predominantly manifested as hyperdense lesions or hyperintense signal shadows in the basal ganglia region, which serve as pivotal diagnostic and differential diagnostic markers for this condition. Importantly, the reversibility of these imaging features provides critical prognostic insights and guides therapeutic strategies.

### Hypotheses

At present, the pathogenesis of DS has not been completely clarified, and everal hypotheses have been proposed in the literature to elucidate its pathogenesis:

1. Neurotransmitter/circuit hypothesis. The neural circuits of the extrapyramidal system may be involved in the pathogenesis of hyperglycemic chorea, In poorly controlled hyperglycemia, striatal GABA stores may be depleted, impairing GABAergic output from the striatum to the internal segment of the globus pallidus (GPi) and substantia nigra pars reticulata (SNr). The resulting thalamic disinhibition increases cortical motor excitability and produces hyperkinetic movements (12). Thus, in diabetic patients with chronically poor glycemic control, oxidative stress in the basal ganglia may further disrupt striatal function, amplifying the risk of chorea, which may result in reduced inhibition of the thalamus by the medial part of the pallidum. Thalamic disinhibition results in the typical dyskinesia, especially for the elderly: the tolerance of brain tissue to metabolic stress is reduced, and neurons in the basal ganglia are susceptible to oxidative damage caused by high blood sugar, leading to striatal dysfunction (13).
2. Dopamine receptor/estrogen hypothesis. Hyperglycemia may directly lead to increased dopamine receptor sensitivity and decreased dopaminergic catabolism, both of which may result in marked changes in dopamine activity in the striatum of the patient. Epidemiologically, DS most often presents between ages 60–80 and shows a female predominance (male:female ≈ 1:2–3), potentially related to hormonal milieu (14, 15), changes in estrogen levels can cause hypersensitivity of dopamine receptors in the striatal region (16). Significantly lower estrogen levels in postmenopausal women may reduce dopamine function in the substantia nigra striatum and increase dopamine receptor sensitivity, thereby increasing the risk of DS. In our case, the elderly female patient improved with glycemic control and a dopamine receptor antagonist, consistent with this mechanism. Estrogen deficiency also explains the prevalence of DS in middle-aged and elderly patients with poor glycemic control (17).
3. Energy-metabolism hypothesis. Hyperglycemia can suppress tricarboxylic acid–cycle flux, shifting neurons toward anaerobic glycolysis (18). In the brain cells of patients in hyperglycemic states, GABA serves as the main source of energy, leading to rapid depletion of GABA, however, GABA cannot be synthesized efficiently, and a decrease in GABA causes a relative hyperactivity of dopamine, leading to decreased muscle tone and increased movement, and ultimately, to extrapyramidal symptoms resembling chorea. It is worth noting that most patients with DS present with chorea symptoms on one side, and this theory cannot fully explain the cause of one side of the disease (19). PET/CT studies in hemichorea–hemiballismus (HC-HB) associated with hyperglycemia show reduced glucose metabolism in the contralateral striatum, supporting a focal energy-metabolism disturbance (20, 21).
4. Vascular-injury hypothesis. This theory includes hemorrhagic and ischemic injuries, and some scholars believe that the high signals on the T1-weighted MR images of patients with DS are caused by ischemic injuries (22). However, some scholars have also found trace hemorrhage in the lesions of patients with DS. However, this hemorrhage was different from typical cerebral hemorrhage in that the hematoma was confined to the region of the putamen and the nucleus accumbens and did not compress the internal capsule and surrounding structures, and no edema or occupying effect was observed (23).

In summary, the pathogenesis of DS encompasses neuropathy and metabolic disorders triggered by hyperglycemia, as well as the effects of gender and hormone levels. Since the pathogenesis of DS has not yet been fully elucidated, current treatment strategies are centered on controlling blood glucose levels and symptomatic treatment of specific symptoms.

### Management

The management of DS primarily revolves around two fundamental therapeutic strategies: glycemic regulation and symptomatic management. Glycemic control is the cornerstone of therapy given the strong association between hyperglycemia and symptom onset (24). Interventions include medical nutrition therapy, oral antihyperglycemic agents, and/or insulin (basal–bolus or continuous subcutaneous insulin infusion) to achieve and maintain normoglycemia. Clinicians should develop personalized glycemic control protocols through comprehensive patient evaluation, accompanied by regular glucose monitoring to facilitate timely therapeutic adjustments (25). Concurrently, symptomatic intervention constitutes an essential therapeutic pillar, particularly targeting choreoathetoid manifestations. Dopamine receptor antagonists are frequently employed for motor symptom modulation, but due to potential adverse reactions, including drug-induced tremors, their administration requires careful dose titration (26). A recommended therapeutic approach involves initiating low-dose regimens with gradual escalation, tailored to individual patient profiles to optimize efficacy while minimizing adverse events (27). Emerging evidence from case studies suggests potential utility of topiramate and valproate sodium, though current data remain limited (28, 29). Non-pharmacological interventions including acupuncture therapy for motor symptom amelioration (30), and physical rehabilitation for enhancing motor coordination and fall prevention may provide adjunctive benefits (31). Comprehensive DS management necessitates dual emphasis on etiological control through sustained normoglycemia and targeted symptom relief, with continuous clinical surveillance enabling dynamic treatment optimization. Notably, while hyperglycemia represents the principal pathogenic trigger, paradoxical reports of hypoglycemia-associated chorea warrant clinical vigilance (32). Favorable outcomes depend on patient education and adherence to medications, systematic glucose monitoring, and maintenance of near-normoglycemia to prevent recurrence.

### Prognosis

Most patients with DS achieve complete symptomatic remission within 12–16 weeks after glycemic stabilization, and neuroimaging abnormalities usually resolve over the same interval. About 20% have persistent symptoms lasting months to years and may require prolonged dopamine receptor antagonist therapy (33). Recurrence correlates strongly with poor glycemic control, with cohort estimates of 10–15% (34). Although DS itself is not associated with increased mortality, outcomes may be adversely influenced by severe metabolic derangements and coexisting cardiovascular or cerebrovascular disease (14).

### Summary

DS is a relatively rare disease, necessitating heightened clinical awareness due to its potentially severe motor manifestations. The management of DS primarily revolves around strict glycemic control, achievable through insulin therapy as the cornerstone of treatment. In cases of severe choreiform movements, Dopamine D_2_ receptor antagonists (e.g., haloperidol) or VMAT2 inhibitors (e.g., tetrabenazine) are commonly used for symptomatic relief (8). Most patients have a favorable course, with gradual improvement or complete resolution of symptoms after optimization of glucose control.

As practicing clinicians, it is imperative to integrate multimodal imaging findings with clinical manifestations for accurate neurologic differential diagnosis, thereby avoiding diagnostic pitfalls. This approach not only ensures timely initiation of appropriate management but also significantly impacts treatment outcomes and prognosis. Clinicians should remain alert to atypical presentations and systematically exclude alternative hyperglycemia-related or concurrent conditions—such as intracerebral hemorrhage, ischemic stroke, and metabolic encephalopathy—through comprehensive evaluation (35).

## Patient perspective

Both patients were unable to provide detailed perspectives during acute illness due to age and altered mental status. Following recovery, patients and families expressed satisfaction with care and outcomes, particularly the resolution of involuntary movements.

## Data Availability

The original contributions presented in the study are included in the article/supplementary material. Further inquiries can be directed to the corresponding author.
